# COVID Pandemic Impact on Nevada Adult Protective Services

**DOI:** 10.1093/geroni/igab046.332

**Published:** 2021-12-17

**Authors:** Holly Ramsey-Klawsnik

**Affiliations:** Klawsnik & Klawsnik Assoc & Consultant, Nevada Adult Protective Services Program, Canton, Massachusetts, United States

## Abstract

Empirical data regarding Covid pandemic impact on the Nevada Adult Protective Services (APS) Program clients, casework, and staff was gathered and analyzed as part of a multi-faceted program evaluation. Key findings include: 66% of the staff agreed or strongly agreed that the pandemic made their jobs more challenging. Respondents reported Covid-related challenges faced by clients, the program, and themselves as social workers serving older and vulnerable adults. Adverse client impacts observed included increased social and emotional isolation, loss of housing, exacerbation of symptoms of mental illness, necessary services being cut from clients subsequent to testing Covid positive, and fear and reluctance to allow needed visiting service providers, such as home health aides, into their homes. We will discuss the implications of the findings on APS services and clients, and on related health and human services designed to promote the wellness and independence of older and vulnerable adults.

